# The Value of Contemplative Practices: A Mixed Methods Approach Exploring Associations between Resilience and Experiences of the COVID-19 Pandemic among Older Adults

**DOI:** 10.3390/ijerph191610224

**Published:** 2022-08-17

**Authors:** Grace Achepohl, Catherine Heaney, Lisa G. Rosas, Jessie Moore, Tia Rich, Sandra J. Winter

**Affiliations:** 1 Stanford Prevention Research Center, Stanford University, 3180 Porter Drive, Palo Alto, CA 94304, USA; 2 Department of Psychology, Stanford University, Building 420, 450 Jane Stanford Way, Stanford, CA 94305, USA; 3 Department of Epidemiology and Population Health, Stanford University, Alway Building, 300 Pasteur Drive, Stanford, CA 94305, USA; 4 Department of Medicine, Division of Primary Care and Population Health, Stanford University, 1265 Welch Road, Stanford, CA 94305, USA; 5 Senior Coastsiders, 925 Main Street, Half Moon Bay, CA 94019, USA

**Keywords:** older adults, contemplative practices, resilience

## Abstract

The aim of this study was to explore the association between resilience and experiences of the COVID-19 pandemic among older adults. We used a sequential explanatory mixed methods study design to recruit older adults who spoke English and were 60 and above during the pandemic. Survey data investigated older adults’ resilience, post-traumatic growth, well-being, and demographics. Extreme case purposeful sampling of their resilience score was used to select interviewees. Qualitative data sought to understand the relationship between resilience and how older adults responded to the COVID-19 pandemic. Exploring the relationship between resilience (well-being in the face of challenge) and one’s experience of the COVID-19 pandemic revealed that participants categorized as having high resilience had long held behaviors of contemplative practices that helped them effectively adapt to the COVID-19 pandemic. As we continue to face global challenges, we must redefine care, guide interventions, and promote healthy aging by incorporating contemplative practices into the lives of older adults.

## 1. Introduction

The COVID-19 pandemic created global disruption. Older adults were likely to experience multiple challenges such as increased risk of their own and their peers’ morbidity and mortality, as well as social isolation and loneliness [1,2,3]. Paired with increased ageism, discourse grew about a possible mental and physical health crisis among older adults [4,5,6]. To the contrary, preliminary research showed older adults’ well-being was as high as, or even higher than, in years prior to the pandemic [7]. One possible explanation for this is that older adults exhibited higher levels of resilience.

When older adults are facing adverse events, resilience offers a multitude of protective factors, such as independence and grit, while moderating their experience of community belonging, social participation, and trauma exposure [8,9,10]. For this study, we defined resilience on a whole person level as “the capacity to maintain, or regain, psychological well-being in the face of challenge” [11]. With historical consensus that resilience is “a dynamic process”, resilience theory emphasizes the possibility of bidirectional adaptation within the context of adverse events such as the COVID-19 pandemic [8,12]. Given that the COVID-19 pandemic was an adverse event that impacted the dynamic process of resilience in older adults, it is important to understand the association between resilience and experiences of the COVID-19 pandemic among older adults, yet very little about this relationship and the diversity within it exists.

Historically, contemplative practice behaviors (meditation, prayer, breath awareness, gratitude, and compassion) have been known to cultivate physical and psychosocial health benefits including resilience. Vago and Silbersweig put forth the Self-Awareness, Self-Regulation, and Self-Transcendence (S-ART) theoretical model to represent the three main mechanisms by which contemplative practices can enhance resilience [13]. Extensive evidence derived from decades of behavioral and neuroscientific findings on contemplative practices has established that they strengthen self-awareness and mindfulness, as well as self-regulation and distress tolerance [14,15,16,17,18,19,20,21]. Additional research indicates that mindfulness may engage emotion-regulation processes that improve healthy lifestyle behaviors [22,23,24,25,26,27,28,29].

Research on the self-transcendence component of the S-ART model indicates that it includes a quality of equanimity—an openness and ability to be present to what is happening in current circumstances with an even-minded state or dispositional tendency toward all experiences and objects, regardless of their affective valence (pleasant, unpleasant, or neutral) [30,31,32,33,34]. Equanimity has been described as both reduced cognitive avoidance as well as automatic affective processing and evaluative judgmental reactions. Thus, equanimity contributes to the ability to maintain calmness in the face of provoking stimuli [30,35]. Furthermore, the implications of cultivating equanimity during the COVID-19 pandemic has been explored in terms of its role in fostering adaptive coping and holistic well-being [36].

Regarding research on contemplative practices among older adults, a recent meta-analysis on the effects of mindfulness meditation on depression in older adults (71.8  ±  5.2 years old) reported mindfulness meditation interventions significantly reduced depressive symptoms in older adults [37]. Mindfulness-based stress reduction programs have also been shown to reduce self-reported loneliness and pro-inflammatory gene expression in older adults [38]. Similarly, a meta-analysis on the effects of meditation and mind–body exercises on older adults’ cognitive performance found improvements to cognition for older adults aged 60 years or above [39].

The COVID-19 pandemic provided a unique opportunity to test the benefits of contemplative practice behaviors in the conditions of a global pandemic [40,41,42]. Furthermore, given that a multitude of global challenges continue to impact older adults, the possible benefits of resilience research extend far beyond the COVID-19 pandemic. 

We used a sequential explanatory mixed methods study design to investigate the association between resilience and the experiences of the COVID-19 pandemic among 61 older adults (aged 60–94 years) living in Northern California.

## 2. Materials and Methods

### 2.1. Participants

We recruited English-speaking adults aged 60 and older living on the San Mateo County Coast of California in the United States who completed a survey either online or using pen and paper. Participants were recruited with the assistance of a local non-profit senior center. Recruitment methods included announcements in an online newsletter sent to approximately 600 people, flyers distributed to approximately 150 home delivered meal participants, and posts on NextDoor and Facebook. An invitation to participate in the study was also distributed to a manufactured home community of residents aged 55 and older (approximately 360 households) and to a low-income senior housing complex (312 units). Participation was voluntary. Surveys with insufficient data (6) and duplicates (2) were excluded. The final sample included 61 individuals who were representative of the ethnic composition of the study location. The San Mateo County Coastal region is primarily white and female (79%; 54%) [43]. On the Coast, 17.7% of the population is over the age of 65 and 49.2% is over the age of 55 [44].

### 2.2. Procedure

This study was approved by the Stanford University Institutional Review Board and the Stanford COVID-19 Clinical Review Panel.

To understand the association between resilience and older adults’ experiences during the COVID-19 pandemic, we conducted a two-phase sequential explanatory mixed methods study which allowed researchers to further understand quantitative findings and explore an older adult community from a holistic perspective. We defined one’s experience of the COVID-19 pandemic broadly as any life change that was directly or tangentially related to the COVID-19 pandemic (e.g., increased traffic by the beach or inability to see family members). The first phase consisted of a quantitative survey completed either online (*n* = 35) or using pen and paper (*n* = 26). Qualtrics was used to capture the data [45]. The survey took approximately 10 min to complete and included four different sections of questions: the Connor Davidson Resilience Scale (CD-RISC 10), the Post-Traumatic Growth Inventory Short Form (PTGI-SF), National Council on Aging’s Well-being Assessment (NCOA AWA), and demographic questions [46,47,48,49]. Resilience, as measured on the CD-RISC 10 scale, was the primary outcome. The survey was open between 8 April and 18 June 2021.

**Figure 1:**
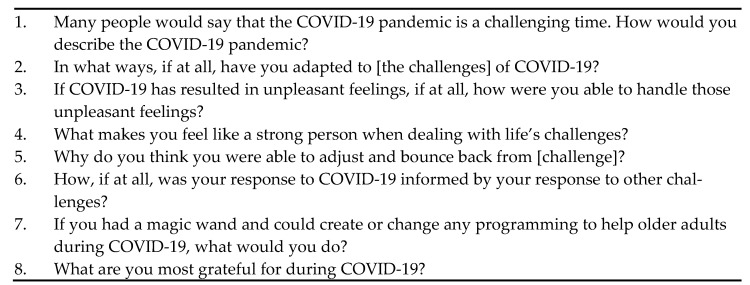
These eight questions were used as the interview guide to structure interviews.

The second phase consisted of in-depth, semi-structured interviews conducted on a sub-sample of participants (*n* = 12, 20%). Interviewees were selected by extreme case purposeful sampling of their CD-RISC 10 score. We contacted 10 individuals categorized as having low resilience and seven individuals categorized as having high resilience via phone and email with the intention of interviewing six individuals per group. We reached out to more individuals categorized as having low resilience because they were harder to contact. One low resilience participant declined to be interviewed, three could not be reached. Six interviewees categorized as having low resilience and seven interviewees categorized as having high resilience were interviewed (one of the seven high resilience interviews was excluded from analysis after researchers became aware that the participant had taken the survey twice, once online and once using pen and paper). All interviews were conducted by the principal investigator (a female graduate student (MS) with expert training and supervision). The interviewees were informed of the nature of the interviewer’s graduate work and had no prior relationship with the interviewer. Interviews occurred between 13 May and 22 June 2021, via phone or Zoom, lasted 41–96 min, were audio-recorded, and transcribed verbatim. Field notes were made after the interview. The interview guide consisted of eight open-ended questions on challenge, adaptation, unpleasant feelings, strength, historical influence, gratitude, and programmatic offerings (Figure 1). Interviews concluded after 12 interviews, a predetermined stopping point which is representative of approximately 20% of the overall sample.

### 2.3. Measures

#### 2.3.1. Resilience

Resilience was assessed with the 10-item CD-RISC score (Cronbach’s alpha = 0.90) which has been validated in older adults and measures the ability to cope with adversity [46,50]. Based on their experience in the past month, participants responded to each item on a 5-point Likert scale from 0 (not true at all) to 4 (true nearly all the time). No items were reverse scored, and all 10 items were summed. Possible scores ranged from 0 to 40.

#### 2.3.2. Post-Traumatic Growth Inventory Short Form

A participant’s ability to see the ‘silver lining’ (SL) was measured by the validated 10-items PTGI-SF (Cronbach’s alpha = 0.86) [47,51]. Based on their experiences over their lifetime, participants responded to each item on a 6-point Likert scale from 0 (I did not experience this change as a result of my challenge) to 5 (I experienced this change to a very great degree as a result of my challenge). A factor analysis conducted by Stanford WELL for Life on 265 older adults aged 60 and above living in Northern California found PTGI-SF items could be described in one factor [52]. No items were reverse scored, and the 10 items were reported independently as well as averaged to an ‘Overall Score.’ Possible scores ranged from 0 to 5.

#### 2.3.3. Well-Being

Well-being was assessed with the 8-item National Council on Aging’s Adult Well-being Assessment (NCOA AWA), a community designed self-administered survey specifically for older adults, that measured characteristics such as mental health, physical health, financial health, social and emotional support, and isolation [49]. Based on their current experience or predicted 2-year experience, participants responded to each item using a ladder or Likert-like scale (ranging from 5 to 11 points). One item was reverse scored, and all 8 item responses were reported separately in categories of thriving, surviving, and suffering that were predetermined by the assessment scoring guide. For example, one question asked, ‘In general, how would you rate your physical health?’ with responses ranging 5 (Excellent) to 1 (Poor). Individuals who rated their physical health as a five or four were considered thriving, three were considered surviving, and two or one were considered suffering. Another question asked, ‘How often do you feel lonely or isolated from those around you?’ with responses ranging from 5 (Always) to 1 (Never). Individuals who rated their feelings of isolation as a one or two were considered thriving, three were considered surviving, and four or five were considered suffering. For more information on scoring, please reference the scoring guide [49].

### 2.4. Analytic Strategy

#### 2.4.1. Quantitative Analysis Procedure

Quantitative analyses were conducted in R [53]. To separate the sample for extreme case purposeful sampling and explore the association between background characteristics and resilience, we categorized participants into high and low resilience groups and calculated descriptive statistics such as mean and standard deviation. Categorization of participants into high and low resilience groups occurred based on the mean 10-item CD-RISC score (31.1 ± 6.3) for older adults in the larger U.S. population [54]. Low resilience was defined by a 10-item CD-RISC score as below 31.1, the published mean score in older adults. High resilience was defined by a 10-item CD-RISC score above 31.1. Resilience groups were compared using chi-squared and Fischer exact tests to determine statistically significant differences. To explore the association between the PTGI-SF and resilience, we compared the means of the PTGI-SF items and overall score across high and low resilience groups using a T-test when normally distributed and a Wilcoxon rank-sum test when the normality assumption was not met. Resilience scores were compared across the AWA-defined well-being groups using a one-way ANOVA. A *p*-value less than 0.05 was considered significant.

#### 2.4.2. Qualitative Analysis Procedure

We used a hybrid qualitative analysis approach [55]. Prior to coding the interviews in NVivo, the principal investigator deidentified interviews, scanned interviews, identified themes, and created a codebook [56]. No interviews were repeated, nor transcripts returned. Multiple codes were allowed and encouraged. To ensure coding reliability, the principal investigator and another individual with qualitative analysis training and experience independently coded three interviews using an initial codebook. Subsequently, they met to discuss and reconcile differences and updated the codebook accordingly. A second round of independent coding and discussion occurred on a fourth interview prior to finalization of the codebook. The principal investigator coded all 12 interviews using the updated codebook. Etic concepts and emic themes contributed to the 16 final codes and 26 final subcodes.

## 3. Results

### 3.1. Quantitative Analysis

Table 1 depicts the demographic characteristics of participants categorized as having high and low resilience. Among the 61 older adults who provided valid data, the majority identified as white females (92%; 74%) and were aged 70–79 years old (39%; range = 60–94). Eleven participants (18%) had two or more difficulties with activities of daily living such as hearing, seeing, remembering, using stairs, dressing, or bathing, and running errands alone. No statistically significant difference in demographic characteristics was observed between participants categorized as having high and low resilience.

Comparing PTGI-SF data for participants who scored above (high resilience) and below (low resilience) the mean population average CD-RISC score, participants categorized as having high resilience were significantly more likely to report that they changed their priorities, had a greater appreciation for life, closeness with others, and knew they could better handle difficulties because of challenges (Table 2). There were no statistically significant differences between participants categorized as having high and low resilience scores regarding spiritual matters, religious faith, finding a new path and learning about how wonderful people are as a result of challenges.

Analysis of the NCOA AWA data revealed that participants categorized as suffering had significantly lower resilience scores than those who were thriving across all eight domains of well-being (life satisfaction, life optimism, physical health, mental health, financial well-being, social and emotional support, meaning and purpose in life, and social isolation and loneliness) (Table 3). ‘Life Optimism’ and ‘Meaning and Purpose in Life’ had insufficient data to report on individuals categorized as suffering.

### 3.2. Qualitative Analysis

#### 3.2.1. Participants

Table 4 depicts the demographic characteristics of interviewees. Among the 12 older adults who were interviewed, all were white, and three-quarters were women. The largest group of participants were aged 80+ years old (42%; range = 60–94). Three interviewees (25%), all of whom were categorized as having low resilience, had two or more difficulties with activities of daily living such as hearing, seeing, remembering, using stairs, dressing, or bathing, and running errands alone.

#### 3.2.2. Impact of COVID-19

All 12 interviewees discussed how COVID-19 had negative, neutral, and positive impacts on their lives. For interviewees categorized as having high resilience an example of a negative impact is Participant F (70–79, male) who spoke of unpleasant feelings he experienced “around the dissemination of misinformation.” An example of a neutral impact is Participant B (70–79, female) who said, “I know your deal is on COVID, but what troubled me most over this past year was the Black Lives Matter stuff.” An example of a positive impact is Participant D (80+, female), who lived alone and said:

“*For years I was a book indexer and it’s a very solitary profession. You don’t have a lot of people running around you all the time and so I’m used to being alone more than a lot of people, I guess and I’m comfortable with it.*”

For interviewees categorized as having low resilience an example of a negative impact is Participant E (60–69, female) who said she “found it challenging at first…I found it confining, um I found that I really had to find almost a new way to reinvent myself.” An example of a neutral impact is Participant C (80+, female), who lived alone and said, “it really didn’t affect me because I don’t leave my house.” An example of a positive impact is Participant A (80+, female) who said:

“*COVID, in a way, I guess was a relief, sort of an excuse to continue with my failure to meet challenges … There were no challenges to meet other than um just getting used to the idea that, I was, even more restrictive than usual.*”

#### 3.2.3. Contemplative Practices and Spirituality

Interviewees categorized as having high resilience were more likely than those categorized as having low resilience to mention contemplative practices, including but not limited to meditation, breath awareness, and gratitude practice, as a coping mechanism throughout the pandemic (4:1 interviewees). Instead of allowing the uncertainty of the pandemic to rattle herself, Participant G (60–69, female), categorized as having high resilience, spoke to how she grounded herself in contemplative practice:

“*Well, I’ve been doing [contemplative practices] for maybe 11, 12 years now. So, I already had, which I’m so grateful for, the experience of knowing that nothing is predictable, that things always change. Like I was saying at the beginning, we always think things are going to be predictable. We never think that something, I mean a bomb could drop tomorrow or in the next moment. We don’t know what’s going to happen.*”

Although meditation was not as common amongst interviewees categorized as having low resilience, Participant E (60–69, female), categorized as having low resilience, found similar benefits, “I have been a meditator for years and during COVID I was really grateful for it.”

Interviewees categorized as having high resilience were more likely than those categorized as having low resilience to mention spirituality, or the belief in something beyond oneself which includes but is not limited to organized religious activities, as a historical coping mechanism that they used throughout the pandemic (6:2 interviewees). One interviewee, categorized as having high resilience, spoke of spirituality as being her source of strength when dealing with life’s challenges:

“*I really think having a church association, a faith of some kind, gives you more strength to weather storms… I really feel like a lot of times, especially after my husband died, you know, you pray for strength and um I don’t know, I feel like I’m a stronger person just because I believed and I’m going to be OK and that life is going to go on and it’s just one foot in front of the other and you try to do the best you can*” *Participant D (80+, female)*.

#### 3.2.4. The Impact of Meditation and Contemplative Practices on COVID-19

Interviewees categorized as having both high and low resilience mentioned the benefit of longstanding meditation and contemplative practices on their experience of the COVID-19 pandemic. When asked how they coped with the frustration of COVID-19, Participant B (70–79, female), categorized as having high resilience, spoke of the importance of meditation:

“*Mostly it’s through my meditation because my meditation takes me back to removing all the illusions of what’s going on, because none of it is real, and the real part is what’s inside of you. And if you keep that healthy and if I keep in touch with myself, that’s the vibration I’m sending out and that’s all I’ve been doing.*”

Participant E (60–69, female), characterized as having low resilience, shared similar sentiments:

“*You know, when I do stuff sometimes, I just start to feel really anxious. It’s just like pay attention to your breath, you know? And that really works for me. I would say I have been a meditator for years and during COVID I was really grateful for it.*”

#### 3.2.5. Physical Limitation and Additional Findings

Although most interviewees (*n* = 9) mentioned some sort of physical limitation that was present during the pandemic, interviewees categorized as having low resilience mentioned the negative impact at a greater frequency (40:13 times) and tended to mention more complex chronic illnesses. These interviewees mentioned difficulty in obtaining groceries and feeling unsafe socializing due to their compromised state. Interviewees categorized as having low resilience were more likely than interviewees categorized as having high resilience to mention the benefits of technology (5:2 interviewees).

Interviewees were asked “If you had a magic wand and could create or change any programing to help older adults during COVID-19, what would you do?” Responses highlighted the need for training in contemplative practices, resuming in person gatherings, increased technology and COVID-19 literacy education, and more advocacy to improve accessibility.

## 4. Discussion

This study allowed us to achieve our aim of listening to and learning about the association between resilience and the COVID-19 pandemic amongst older adults where we found that among our participants, many of those categorized as having high resilience had long held behaviors of contemplative practices that helped them effectively adapt to the COVID-19 pandemic. We used a sequential explanatory mixed methods study design to recruit 61 participants across a 34-year age span (aged 60–94 years) who had significantly different lived experiences. We found the COVID-19 pandemic to have negative and positive impacts on all interviewee’s lives, regardless of resilience.

Instead of viewing contemplative practices as something that was developed because of the pandemic, the results show it to be a protective factor that benefited individuals categorized as having high resilience during the COVID-19 pandemic. Our quantitative survey asked participants if they had experienced a change in their spiritual or religious practices because of a recent challenge. Contrary to expectations, participants reported no change in their spiritual or religious practices. In-depth interviews uncovered that most participants categorized as having high resilience had long held behaviors of contemplative practices that helped them effectively adapt to the COVID-19 pandemic. In addition, we found a strong relationship between resilience and well-being as seen in Table 3. This aligns with the S-ART model and previous research on the positive effect contemplative practices have on resilience and well-being, as well as the current literature calling for meditation and mindfulness during times of crisis [36,42,57,58,59,60].

Practically, the findings of this study indicate a need for providers to encourage older adults to participate in activities that build resilience such as cultivating contemplative practice behaviors [42,61]. The study site is in the process of implementing a yearlong contemplative practice behavior development program via Zoom and in person that is funded through the county government and delivered by contemplative practice experts. It will be available free of charge to older adults and other community members. A resource booklet will be developed to assist interested individuals in finding more information about contemplative practices.

Although our study looked at resilience in the face of COVID-19, building older adults’ resilience through contemplative practices is vital for so much more. Improving resilience through contemplative practices may help older adults adapt and cope with a multitude of global challenges such as pandemics, structural inequality, racism, natural disasters, gun violence, and climate change [62].

### 4.1. Strengths and Limitations

To recruit participants of differing socioeconomic status, technological literacy, and ages, we worked with the staff at a local senior center to distribute the flyer to their home delivered meals participants, in their newsletter, to interested participants and local community-based partners (including but not limited to a manufactured home community of residents aged 55 and older and to a low-income senior housing complex). We posted the recruitment flyer on NextDoor and Facebook. We also made the survey accessible in both online and pen and paper formats to cater to different technological literacy levels. We designed the survey specifically for older adult accessibility by increasing text size, being mindful of color usage, and including images. During the interview phase, we paid particular attention to the outreach of individuals categorized as having low resilience to ensure proper representation and offered interviews via Zoom and phone to ensure all individuals could participate.

Notwithstanding these strengths, there were some limitations. The sample size was small, primarily white and female, and therefore not broadly generalizable. At the same time, the predominantly white sample represented the Northern California community in which the study was conducted where 79% of the population is white [43]. Due to language limitations, we were unable to include monolingual Mandarin or Spanish speakers which limited findings to English language speakers. Another study limitation is that all quantitative data was collected through self-reported measures which means there was inherent subjectivity. However, this research serves as a starting point for investigating the relationship between contemplative practice behaviors and resilience among older adults. It may also be argued that there was social desirability bias as the study was being conducted in partnership with a community organization and participants may have wanted to please the community organization and their peers by participating. However, the principal investigator, who conducted all interviews, was not working for the community organization nor known to participants. Finally, there is a chance that data is reported as overly significant due to the multiple comparisons problem and therefore should be interpreted cautiously.

### 4.2. Future Direction

Future research should specifically investigate the benefits of contemplative practices during other large-scale challenges such as future pandemics, structural inequality, racism, natural disasters, gun violence, and climate change. Future research is also recommended to investigate resilience developed through contemplative practices among populations not well represented in this study including males, people of color, and indigenous people.

As we continue to face global challenges, we must redefine care, guide interventions, and promote healthy aging by incorporating contemplative practices into the lives of older adults. On a community level, non-profit organizations should be intentional about cultivating contemplative practices. On a provider level, physicians should consider asking older adults about their contemplative practices and encouraging them to continue developing their resilience.

## 5. Conclusions

We found that among our participants, many of those categorized as having high resilience had long held behaviors of contemplative practices that helped them effectively adapt to the COVID-19 pandemic. The present study thus contributes to the existing academic literature by developing an understanding of the benefits of contemplative practice during the COVID-19 pandemic amongst a Northern California community of older adults. We must recognize the benefit of contemplative practices and build a system to ensure their use.

## Figures and Tables

**Table 1:** Demographics.

Characteristic	Total (*n* = 61)	High Resilience * (*n* = 28)	Low Resilience * (*n* = 33)	*p*-Value ^g^
	% (*n*)	% (*n*)	% (*n*)	
Gender				0.84
Female	73.8% (45)	75.0% (21)	72.7% (24)	
Race ^a^				0.09
White	94.9% (56)	88.9% (24)	100.0% (32)	
Other ^b^	5.1% (3)	11.1% (3)	0.0%	
Living Situation				0.77
Alone	57.4% (35)	53.6% (15)	60.6% (20)	
With Another Person ^c^	42.6% (26)	46.4% (13)	39.4% (13)	
Survey Type				0.82
Online Survey	57.4% (35)	60.7% (17)	54.5% (18)	
Paper and Pen Survey	42.6% (26)	39.3% (11)	45.5% (15)	
Age				0.52
60–69	24.6% (15)	28.6% (8)	21.2% (7)	
70–79	39.3% (24)	42.9% (12)	36.4% (12)	
80+	36.1% (22)	28.6% (8)	42.4% (14)	
Monthly Income ^d^				0.08
Less than USD 2000	25.9% (14)	25.9% (7)	25.9% (7)	
USD 2000–3999	31.5% (17)	18.5% (5)	44.4% (12)	
USD 4000+	42.6% (23)	55.6% (15)	29.6% (8)	
Provided Care to Another ^e^				0.64
Yes	46.7% (28)	51.9% (14)	42.4% (14)	
No	53.3% (32)	48.1% (13)	57.6% (19)	
Difficulties with Activities of Daily Living ^f^				0.64
Zero	60.7% (37)	64.3% (18)	57.6% (19)	
One	21.3% (13)	17.9% (5)	24.2% (8)	
Two+	18.0% (11)	17.9% (5)	18.2% (6)	

*Note.* * High resilience is defined by a 10-item CD-RISC score above 31.1. Low resilience is defined by a 10-item CD-RISC score at or below the published mean score in older adults of 31.1. ^a^ Two data points are unknown. ^b^ Includes American Indian or Alaska Native, Asian, Black or African American, and Native Hawaiian or other Pacific Islander. ^c^ Includes spouse only, spouse and children, children only, other family, assisted living, and other. ^d^ Seven data points are unknown. ^e^ One data point is unknown. ^f^ Includes difficulty hearing, seeing, remembering, using stairs, dressing or bathing, and running errands alone. ^g^ *p*-value is for χ^2^ test except race, monthly income, and activities of daily living where Fisher exact test was used.

**Table 2:** PTGI-SF comparisons of participants with high and low resilience.

Question	Total (*n* = 61)		High Resilience * (*n* = 28)		Low Resilience * (*n* = 33)		*p*-Value ^b^
	Mean	SD	Mean	SD	Mean	SD	
1. I changed my priorities about what is important in life.	3.7	1.11	4.0	0.98	3.4	1.16	0.04
2. I have greater appreciation for the value of my own life.	3.9	0.97	4.1	0.97	3.7	0.92	0.02
3. I am able to do better things with my life.	3.6	0.85	3.9	0.73	3.3	0.85	0.05
4. I have a better understanding of spiritual matters.	3.3	1.54	3.5	1.42	3.1	1.63	0.41
5. I have a greater sense of closeness with others.	3.5	1.03	3.8	1.02	3.2	0.94	0.01
6. I established a new path for my life.	3.3	1.50	3.5	1.53	3.0	1.47	0.23
7. I know better that I can handle difficulties.	3.8	0.90	4.1	0.82	3.5	0.87	<0.01
8. I have a stronger religious faith.	2.6	1.85	2.6	1.85	2.5	1.87	0.69
9. I discovered that I’m stronger than I thought I was.	3.7	1.23	3.9	1.34	3.5	1.12	0.06
10. I learned a great deal about how wonderful people are.	3.8	1.10	4.0	1.05	3.6	1.12	0.12
Overall Score ^a^	3.5	0.82	3.8	0.81	3.3	0.78	0.02

*Note.* * High resilience is defined by a 10-item CD-RISC score above 31.1. Low resilience is defined by a 10-item CD-RISC score at or below the published mean score in older adults of 31.1. ^a^ Overall Score is an average of the participants other responses. ^b^ *p*-value is for Wilcoxon rank-sum test except 3, 5, 6, and Overall Score where a *t*-test was used.

**Table 3:** Resilience scores by well-being domain and response category.

Question	Thriving			Surviving			Suffering			*p*-Value ^a^
	*n*	Mean	SD	*n*	Mean	SD	*n*	Mean	SD	
Life Satisfaction	51	31.8	4.81	8	23.9	4.39	-	-	-	<0.01
Life Optimism ^b^	44	32.2	5.14	10	28.2	2.97	6	20.5	2.74	<0.01
Physical Health	31	33.3	4.84	18	28.9	3.98	12	24.8	5.91	<0.01
Mental Health	40	32.6	4.63	13	27.2	5.74	8	24.0	4.63	<0.01
Financial Well-Being	44	31.3	5.58	11	29.6	5.32	6	24.8	6.24	0.03
Social and Emotional Support	38	33.2	3.87	18	26.6	5.33	5	22.4	5.77	<0.01
Meaning and Purpose in Life	41	32.6	4.76	19	26.2	4.83	-	-	-	<0.01
Social Isolation and Loneliness	25	33.0	4.60	24	28.8	5.62	12	27.8	6.75	<0.01

*Note.* Mean and standard deviation of the 10-item CD-RISC score is presented for each domain and category. The categories of ‘Thriving,’ ‘Surviving,’ and ‘Suffering’ are defined in the NCOA AWA scoring guide. ‘Life Optimism’ and ‘Meaning and Purpose in Life’ had insufficient data to report on individuals classified as suffering. ^a^ All *p*-values are for the one-way ANOVA. ^b^ One data point is unknown.

**Table 4:** Interviewee demographic characteristics.

Characteristic	Total (*n* = 12)
	**% (*n*)**
Gender	
Female	75.0% (9)
Race	
White	100.0% (12)
Other ^a^	0.0% (0)
Living Situation	
Alone	58.3% (7)
With Another Person ^b^	41.7% (5)
Survey Type	
Online Survey	58.3% (7)
Paper and Pen Survey	41.7% (5)
Age	
60–69	33.3% (4)
70–79	25.0% (3)
80+	41.7% (5)
Monthly Income ^c^	
Less than USD 3999	55.6% (5)
USD 4000+	44.4% (4)
Provided Care to Another	
Yes	16.7% (2)
No	83.3% (10)
Difficulties with Activities of Daily Living ^d^	
Zero	41.7% (5)
One	33.3% (4)
Two+	25.0% (3)

*Note.* This table provides baseline demographic characteristics for the 12 interview participants of which six participants were selected from the top of the high resilience survey participants and six participants were selected from the bottom of the low resilience survey participants. ^a^ Includes American Indian or Alaska Native, Asian, Black or African American, and Native Hawaiian or other Pacific Islander. ^b^ Includes spouse only, spouse and children, children only, other family, assisted living, and other. ^c^ Three data points are unknown. ^d^ Includes difficulty hearing, seeing, remembering, using stairs, dressing or bathing, and running errands alone.

## Data Availability

Not applicable.
